# Robust Multi-Frame Super-Resolution Based on Adaptive Half-Quadratic Function and Local Structure Tensor Weighted BTV

**DOI:** 10.3390/s21165533

**Published:** 2021-08-17

**Authors:** Shanshan Liu, Minghui Wang, Qingbin Huang, Xia Liu

**Affiliations:** 1 College of Computer Science, Sichuan University, Chengdu 610065, China; lss366@sicnu.edu.cn (S.L.); liuxia201701@163.com (X.L.); 2 Enrollment and Employment Department, Sichuan Normal University, Chengdu 610066, China; 3 Science and Technology Branch, Southwest Jiaotong University Press, Chengdu 610031, China; swjtuphuangqingbin@163.com

**Keywords:** multi-frame super-resolution, half-quadratic function, local structure tensor, bilateral total variation, preserve the edge information

## Abstract

It is difficult to improve image resolution in hardware due to the limitations of technology and too high costs, but most application fields need high resolution images, so super-resolution technology has been produced. This paper mainly uses information redundancy to realize multi-frame super-resolution. In recent years, many researchers have proposed a variety of multi-frame super-resolution methods, but it is very difficult to preserve the image edge and texture details and remove the influence of noise effectively in practical applications. In this paper, a minimum variance method is proposed to select the low resolution images with appropriate quality quickly for super-resolution. The half-quadratic function is used as the loss function to minimize the observation error between the estimated high resolution image and low-resolution images. The function parameter is determined adaptively according to observation errors of each low-resolution image. The combination of a local structure tensor and Bilateral Total Variation (BTV) as image prior knowledge preserves the details of the image and suppresses the noise simultaneously. The experimental results on synthetic data and real data show that our proposed method can better preserve the details of the image than the existing methods.

## 1. Introduction

Super Resolution (SR) refers to the process of reconstructing a High Resolution (HR) image from a single Low Resolution (LR) image or multiple low resolution images by the software method without modifying the hardware environment. SR is often used in satellite remote sensing [1], medical diagnosis [2] and video surveillance [3] and so forth. According to the number of input low resolution images, it can be divided into Single Image Super Resolution (SISR) and Multi-Frame Super Resolution (MFSR). This paper mainly studies MFSR. 

**Figure 1:**
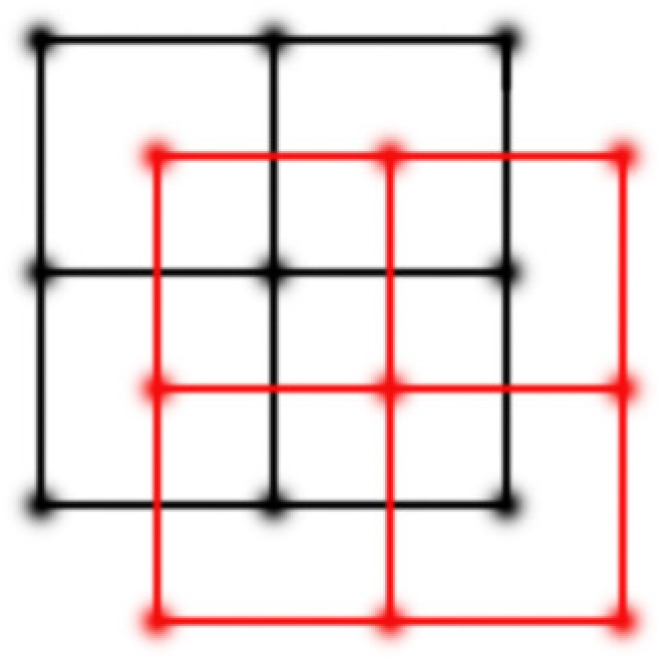
Sub-pixel displacement.

The first MFSR was proposed [4] in 1984, and is based on the frequency domain. The frequency domain method is easy to implement and cheap computationally. However, this method has no way to add image prior knowledge, and can only achieve good results for images without noise and degradation, which is not feasible in practical applications. Therefore, algorithms in the spatial domain are widely studied. There are interpolation-based methods [5,6], reconstruction-based methods [7,8,9,10] and learning-based methods [11,12]. With the popularity of deep learning architectures, many deep learning networks are implemented for MFSR [13,14,15,16]. Learning-based methods need a large number of external training sets; the effect is closely related to the type and number of training sets. Although learning-based SR methods have been hot topics of research in the last few decades, reconstruction-based methods, which do not require training sets, are very popular. The quality of the reconstructed image only depends on our mathematical model and input LR images. This paper mainly studies reconstruction-based MFSR methods that reconstruct an HR image by using the redundancy and supplementary information between multiple LR images in the same scene. In order to find useful information redundancy, an important condition for realizing MFSR is to have sub-pixel displacement as shown in Figure 1. 

Because SR itself is an ill-posed problem, regularization techniques are widely used to solve the minimization problem. The methods of regularization mainly contain the fidelity term and the regularization term. The purpose of the fidelity term is to minimize the observation errors between the reconstructed HR image and the input LR images. The purpose of the regularization term is to make the reconstructed HR image reach a robust state.

The widely-used fidelity terms in the SR are the L1 norm and the L2 norm. Both the L1 norm and the L2 norm have their advantages and drawbacks. The L2 norm can produce lower variance than the L1 norm. However, the L2 norm is sensitive to outliers. M-estimators are explored in MFSR later. For example, the Huber function [17] is proposed for the fidelity term. In [18], the Lorentzian norm is applied to MFSR to increase the robustness. In [19], the performances of the Tukey, Lorentzian and Huber norms are studied concerning outliers. A locally adaptive L1–L2 fidelity norm is also proposed in [20]. Xueying Zeng et al. [8] introduce the adaptive fidelity term based on a new M-estimator half-quadratic function and bilateral edge-preserving (BEP) regularization method. Köhler et al. [9] propose a weighted Gaussian observation model which takes into account the spatial variant noise and weighted bilateral total variation to exploit the sparsity of natural images. Xiaohong Liu et al. [10] use the half-quadratic estimation method to adaptively select the error norm and propose an adaptive bilateral total variation regularization method.

Image prior knowledge is used to regularize the image reconstruction. Tikhonov regularization [21] is one of most commonly used methods. The Total Variation (TV) family, such as Bilateral Total Variation (BTV) [7], is another popular regularization technique. The BTV uses the L1 norm to handle outliers, unlike Tikhonov regularization which is based on the L2 norm.

In recent years, many researchers have proposed a large number of MFSR methods, but MFSR is an ill-posed problem and it is very difficult to reconstruct a satisfactory HR image because the result of MFSR is affected by motion estimation, image registration, unknown blur, noise, and so forth. Even if they are studied separately, each affecting factor is extremely challenging.

In order to simultaneously preserve the image edge and texture details and remove the influence of noise, we propose a new robust MFSR method. In this paper, there are three major contributions to improving the quality of the final reconstructed HR image, as follows:

- We propose a fast and effective method for selecting appropriate LR images, which provide a relatively good input for the reconstruction;
- A novel fidelity term is proposed. We adopt the half-quadratic function as the error norm adaptively. All parameters are automatically adjusted according to observation errors;
- We propose a local structure tensor weighted BTV regularization term. The novel image prior can better simultaneously preserve image details and suppress noise by assigning a certain weight to each pixel according to the local structure tensor information of the image.

The rest of this paper is organized as follows. Section 2 introduces the observation model and the basic framework of MFSR. Section 3 describes our proposed algorithm in detail. Section 4 presents our experimental results on synthetic data and real data. Section 5 summarizes our paper.

## 2. Observation Model and Basic Framework of MFSR

In practical applications, noise, blur, motion and other factors will affect the final SR. Therefore, the observation model of MFSR can be expressed as follows

(1) $$Y_{k}=DB_{k}M_{k}X+n_{k},$$

where $Y_{k}$ represents the *k*th LR image, k=1,2,…,K. The size of $Y_{k}$ is mn×1,  K is the number of LR images. X represents the HR image, the size is rmrn×1,the down sampling factor is r. $M_{k}$ represents the geometric motion matrix (sub-pixel displacement) of the *k*th LR image, the size is mrn×rmrn. $B_{k}$ represents the blur matrix of the *k*th LR image, the size is rmrn×rmrn. D represents the down sampling matrix, the size is mn×rmrn. $n_{k}$ represents the noise of the *k*th LR image, the size is mn×1.

We can simplify Equation (1) by combining $DB_{k}M_{k}$ as a system matrix $W_{k}$ [22], and Equation (1) can be rewritten as follows

(2) $$Y_{k}=W_{k}X+n_{k}.$$

The observation error $r_{k}$ of the LR image $Y_{k}$ can be defined as

(3) $$r_{k}=\left|W_{k}\hat{X}-Y_{k}\right|,$$

where |·| means the absolute value and $\hat{X}$ represents the estimated HR image.

The basic framework of MFSR can be expressed as

(4) $$\hat{X}=\underset{X}{argmin}\left\{\sum_{k=1}^{K}{\left||r_{k}|\right|}_{p}^{p}+\lambda \;\gamma \left(X\right)\right\},\;$$

where γ(X) is the regularization term with respect to X,  p represents $L_{p}$ norm, and λ is the trade-off parameter between the fidelity term and the regularization term.

BTV is calculated simply and is easy to implement, so it is often selected in the regularization term. The formula of BTV can be expressed as

(5) $$\gamma \left(X\right)=\sum_{n=-P}^{P}\sum_{m=-P}^{P}\zeta^{\left|m\right|+\left|n\right|}{\left\|X-S_{x}^{n}S_{y}^{m}X\right\|}_{1},\;$$

where P represents the size of the sliding window.  ζ is a scaled weight parameter. The range of ζ is 0<ζ<1. $S_{x}^{n}$, $\;S_{y}^{m}$ denotes shifting X by n pixels in the horizontal direction and by m pixels in the vertical direction, respectively.

## 3. Proposed MFSR Algorithm

We first select appropriate LR images from a set of LR frames as the input for MFSR. The overall SR framework of this paper is to employ a coarse to fine strategy, enlarging LR images to the target image size gradually in order to adapt to the large scale factor. The half-quadratic function as the loss function is used to control residuals between the unknown HR image and LR images for the fidelity term. For the regularization term, we combine the local structure tensor information of the image and the image sparsity as the image prior knowledge to regularize the estimated HR image.

### 3.1. Selecting the Appropriate LR Images and Alignment

Many existing MFSR algorithms input all LR images instead of selecting input LR images. Some LR images not only do not improve the quality of the reconstructed HR image, but also consume more time and increase the computational complexity. In order to improve the practical applications of MFSR, it is necessary to select LR images with appropriate quality. In this paper, we can remove some low quality LR images quickly to SR.

The number of images required for SR is at least twice the square of the magnification factor under the ideal environment according to [23]. The number of appropriate frames K can be expressed as

(6) $$r^{2}\leq K\leq M,$$

where M is the number of all LR images,  r is the amplification factor.

Although there are many quality evaluation methods [24,25,26] for image selection, most of them are computationally intensive. We need a simple and effective method to quickly select appropriate LR images, so we propose a method of minimum variance. The specific steps for selecting appropriate LR images are as follows:

1. Calculate the variance of all LR images. The variance formula of the *k*th LR image $Y_{k}$ is as follows
  (7) $$s_{k}^{2}=\frac{1}{mn}\sum_{x=1}^{m}\sum_{y=1}^{n}{\left(Y_{k}-\bar{Y_{k}}\right)}^{2},\;$$
  where  m,n represents the width and height of the LR image $Y_{k},$ respectively, $\bar{Y_{k}}$ is the mean value of the LR image $Y_{k}$, and the calculation formula is:
  (8) $$\bar{Y_{k}}=\frac{1}{mn}\sum_{x=1}^{m}\sum_{y=1}^{n}Y_{k}$$
2. Calculate the average of the variance of all LR images, which can be expressed as:
  (9) $$\bar{s^{2}}=\frac{1}{M}\sum_{k=1}^{M}s_{k}^{2}.\;$$
3. Calculate the difference between variance per LR image and the average of the variance which can be defined as:
  (10) $$\Delta s=\left|s_{k}^{2}-\bar{s^{2}}\right|.$$
4. Sort Δs and choose small Δs corresponding LR images. Appropriate LR images are adaptively selected combining Equation (6) and small Δs.

This paper uses the optical flow algorithm to register selected LR images.

### 3.2. Proposed Fidelity Term

Our proposed algorithm is based on the maximum a posteriori (MAP) estimation and Bayesian theory, the expression of the estimated HR image can be written as follows:

(11) $$\hat{X}=arg\;\underset{X}{\max}p\left(X|Y_{1},Y_{2},\ldots ,Y_{K}\right)\;=arg\;\underset{X}{\max}\frac{p\left(Y_{1},Y_{2},\ldots ,Y_{K}|X\right)p\left(X\right)}{p\left(Y_{1},Y_{2},\ldots ,Y_{K}\right)}.$$

Since $p\left(Y_{1},Y_{2},\ldots ,Y_{K}\right)$ has no effect on the estimate X, the above formula can be rewritten as:

(12) $$\hat{X}=arg\;\underset{X}{\max}\;p\left(Y_{1},Y_{2},\ldots ,Y_{K}|X\right)p\left(X\right).$$

Let us suppose that each LR image $Y_{k}$ is independent. The Equation (12) can be simplified to

(13) $$\hat{X}=arg\;\underset{X}{\max}\prod_{k=1}^{K}p\left(Y_{k}|X\right)p\left(X\right),\;$$

where $p\left(Y_{k}|X\right)$ represents the conditional probability of the LR image $Y_{k}$. Given the HR image X, p(X) is the prior probability for the HR image X.

The Half-Quadratic (HQ) function is first proposed in [27] as a potential function. The formula is as follows:

(14) $$f\left(x,a\right)=a\sqrt{a^{2}+x^{2}}-a^{2},$$

where a is a positive constant.

**Figure 2:**
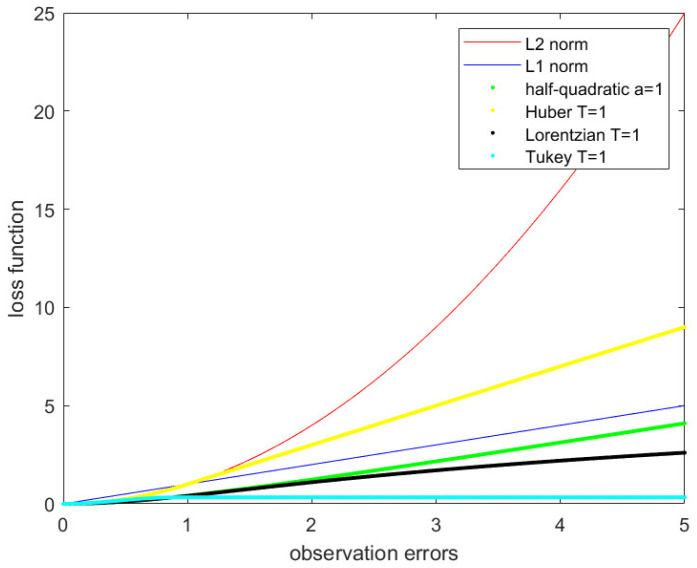
L1 norm, L2 norm, half-quadratic function, Huber function, Lorentzian function, Tukey function.

The L1 norm, L2 norm, half-quadratic function, Huber function, Lorentzian function and Tukey function are shown in Figure 2. We find that the half-quadratic function is close to the L2 norm when observation errors are small and it is close to the L1 norm when observation errors are big. This paper uses the half-quadratic function, which combines the advantages of the L1 norm and the L2 norm as the loss function. The half-quadratic function is strictly convex and twice continuously differentiable to obtain the optimum value easily.

The fidelity term of our robust MFSR by the half-quadratic function is:

(15) $$\hat{X}=\underset{X}{argmin}\left\{\sum_{k=1}^{K}\sum_{i=1}^{I}C\cdot (a_{k}\sqrt{a_{k}^{2}+r_{k,i}^{2}}-a_{k}^{2})\right\},\;$$

where $a_{k}$ is the half-quadratic parameter for the *k*th LR image, $r_{k,i}$ represents the observation error of the *i*th pixel of the *k*th LR image.

**Figure 3:**
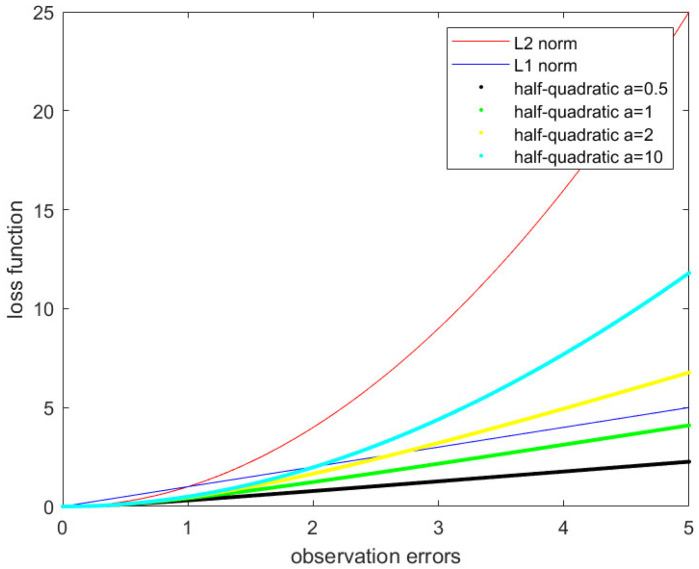
Half-quadratic function with different a values.

Figure 3 shows the performance of the half-quadratic function with different a values. We can see from Figure 3 that the larger the a value is, the closer the half-quadratic function is to the L2 norm. The smaller the a value is, the closer it is to the L1 norm. Therefore, the a value is inversely proportional to observation errors.

The observation error of the *k*th LR image $r_{k}$ can be expressed as:

(16) $$r_{k}=\sum_{i=1}^{I}\left|r_{k,i}\right|,\;$$

where |·| means absolute value, I is the total number of pixels per LR image.

$a_{k}$ can be defined as:

(17) $$a_{k}=\frac{\max\left(r_{k}\right)}{r_{k}},$$

where $\max\left(r_{k}\right)$ represents the maximum value of $r_{k}$.

C is the confidence matrix composed of confidence weights, $C={\left(\beta_{1},\ldots ,\beta_{k},\ldots ,\beta_{K}\right)}^{T}$. For each LR image, the observation error is larger and the corresponding confidence weight should be smaller. So, the confidence weight of the *k*th LR image $\beta_{k}$ can be expressed as:

(18) $$\beta_{k}=\{\begin{array}{c} \frac{\mathrm{mean}\left(r_{k}\right)}{r_{k}}\;\;\;\;\;\;\;\;\;\;\;\;\;\;\;\;\;\;\;\;\;\;\;if\;\left|r_{k,i}\right|\leq c\sigma_{f}^{t},\\ \frac{\mathrm{mean}\left(r_{k}\right)}{r_{k}}\;\cdot \;\frac{c\sigma_{f}^{t}}{\left|r_{k,i}\right|}\;\;\;\;\;\;\;\;otherwise,\;\;\;\;\;\;\;\;\;\;\;\;\;\;\;\;\;\;\;\;\;\; \end{array}$$

where $\mathrm{mean}\left(r_{k}\right)$ represents the mean value of $r_{k}$. c is a positive constant, $\sigma_{f}^{t}$ is the estimate value of the scale parameter in each iteration t to discriminate inliers and outliers adaptively. c is set to 2 in this paper. We use the median absolute deviation (MAD) [28] to estimate $\sigma_{f}^{t}$, the formula can be expressed as:

(19) $$\sigma_{f}^{t}=\sigma_{0}\cdot MAD\left(r^{t-1}|\beta^{t-1}\right),$$

where we set $\sigma_{0}=1.4826$ for the Gaussian distribution.

### 3.3. Proposed Local Structure Tensor Weighted BTV Regularization Term

Due to the BTV regularization term, we cannot distinguish the edge information of the image well. We propose combining the local structure tensor information of the image with the image sparsity as image prior knowledge in order to simultaneously better preserve the edge information of the image and suppress the noise.

The proposed regularization term can be expressed as:

(20) $$\gamma \left(X\right)=\sum_{n=-P}^{P}\sum_{m=-P}^{P}w_{T}\cdot \zeta^{\left|m\right|+\left|n\right|}{\left\|X-S_{x}^{n}S_{y}^{m}X\right\|}_{1}.\;$$

The value of $w_{T}$ depends on the local structure tensor of the image. The local structure tensor can well describe the local structure information of the image, which can be expressed as:

(21) $$T=\left[\begin{array}{cc} I_{x}^{2} & I_{x}I_{y}\\ I_{x}I_{y} & I_{y}^{2} \end{array}\right],$$

where $I_{x},I_{y}$ represents the gradient in horizontal and vertical directions, respectively.

Because the local structure tensor T is a positive semi-definite matrix and has two nonnegative eigenvalues, $\lambda_{1}$ and $\lambda_{2}$ ($\lambda_{1}\geq \lambda_{2}$), the spatial structure information of the image can be divided into three cases: When $\lambda_{1}\approx \lambda_{2}\approx 0$, it means that the gray level change of the image along any direction is very small; it is a flat area. When $\lambda_{1}>\lambda_{2}\approx 0$, it means that the gray change rate of the image along a certain direction at this point is larger; it is an edge region. When $\lambda_{1}\geq \lambda_{2}>0,$ it means that the gray level of the image changes greatly in both vertical directions; the point is a corner.

In order to describe the local structure information of the image well, we construct a local structure tensor weight matrix $w_{T,}$ which is expressed as:

(22) $$w_{T}=\frac{1}{w+\frac{1}{2}e^{-{\left(\lambda_{1}-\lambda_{2}\right)}^{2}}},$$

where w is a fine tuning parameter; we set w=0.5.

As the image prior weight, ζ in Equation (20) related to $X-S_{x}^{n}S_{y}^{m}\;X$ can be calculated adaptively as:

(23) $$\zeta =\{\begin{array}{c} 1\;\;\;\;\;\;\;\;\;\;\;\;\;\;\;\;\;\;\;\;\;\;\;if\;\left|X-S_{x}^{n}S_{y}^{m}X\right|\leq c\sigma_{p}^{t},\\ \frac{c\sigma_{p}^{t}}{\left|X-S_{x}^{n}S_{y}^{m}X\right|}\;\;\;\;\;\;\;\;otherwise,\;\;\;\;\;\;\;\;\;\;\;\;\;\;\;\;\;\;\;\;\;\; \end{array}$$

where c is a positive constant, c is set to 2 in our paper. $\sigma_{p}^{t}$ is obtained from the distribution ${\left|X-S_{x}^{n}S_{y}^{m}X\right|}^{t-1}$ and the image prior weight $\zeta^{t-1}$, the formula similar to $\sigma_{f}^{t}$ is as follows

(24) $$\sigma_{p}^{t}=\sigma_{0}\cdot MAD\left({\left|X-S_{x}^{n}S_{y}^{m}X\right|}^{t-1}|\zeta^{t-1}\right),$$

where $\sigma_{0}=1$ for the Laplacian distribution.

### 3.4. Image Reconstruction

In this paper, the formula of the reconstructed HR image can be written as

(25) $$\hat{X}=\underset{X}{argmin}\left\{\sum_{k=1}^{K}\sum_{i=1}^{I}C\cdot (a_{k}\sqrt{a_{k}^{2}+r_{k,i}^{2}}-a_{k}^{2})+\lambda \cdot \sum_{n=-P}^{P}\sum_{m=-P}^{P}w_{T}\cdot \zeta^{\left|m\right|+\left|n\right|}{\left\|X-S_{x}^{n}S_{y}^{m}X\right\|}_{1}\right\}.\;$$

The regularization parameter λ is used to balance the fidelity term and the regularization term. We use cross validation, which is used by Köhler et al. [9] to determine the value of λ.

There are many optimization algorithms for solving the minimization problem for MFSR. We employ Scaled Conjugate Gradient (SCG) [29] to solve the problem in the Equation (25) in this paper because the convergence of SCG is fast and the SCG algorithm can adjust the step size adaptively. We set the iteration threshold to $10^{-3}$. In the process of optimization, the first-order derivative function of X can be calculated as:

(26) $$f^{\prime }=\sum_{k=1}^{K}\sum_{i=1}^{I}C\cdot a_{k}\frac{W_{k}^{T}\left(W_{k}X-Y_{k}\right)}{\sqrt{a_{k}^{2}+{\left(W_{k}X-Y_{k}\right)}^{2}}}+\lambda \cdot \sum_{n=-P}^{P}\sum_{m=-P}^{P}w_{T}\cdot \zeta^{\left|m\right|+\left|n\right|}\left(I-S_{x}^{-n}S_{y}^{-m}\right)sign\left(X-S_{x}^{n}S_{y}^{m}X\right),$$

where $S_{x}^{-n},S_{y}^{-m}$ is the transpose matrix of $S_{x}^{n},S_{y}^{m}$ respectively. I is an identity matrix.

## 4. Experimental Results and Analysis

We used synthetic data and real data to test our proposed MFSR algorithm. We implemented our method on a laptop computer with Intel(R) Core(TM) i7-8650U CPU and 16 GB RAM, (Intel, Santa Clara, CA, USA). Our algorithm was compared to the following MFSR algorithms: L2 + Tikhonov [21], L2 + BTV [7], L1 + BTV [7], BEP [8], DeepSR [13], IRW [9], and SWHQ + ABTV [10].

### 4.1. Experiments on Synthetic Data

**Figure 4:**
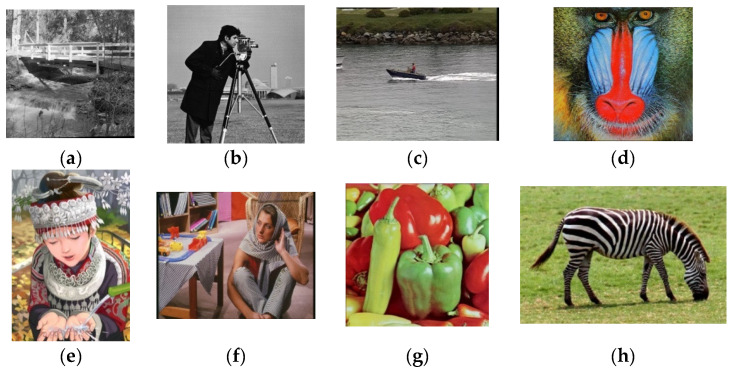
8 HR images for synthetic LR images. (**a**) Bridge. (**b**) Cameraman. (**c**) Coastguard. (**d**) Baboon. (**e**) Comic. (**f**) Barbara. (**g**) Pepper. (**h**) Zebra.

Our proposed method was first measured on synthetic data quantitatively, because the ground truth HR images were available. We used peak-signal-to-noise ratio (PSNR), structural similarity (SSIM) and Information fidelity criterion (IFC) to assess image quality in this paper. We used common HR images to generate synthetic LR images by using the Set 14 dataset [30], shown in Figure 4. We created 30 LR images from one HR image by adding random motion, blur, down sampling and noise. The range of random translations was from −3 to +3 pixels. The range of random rotation angles was from −1° to +1°. Each LR image was blurred by a Gaussian PSF with σ=0.4. We put in mixed noises by Gaussian noise and Poisson noise.

**Figure 5:**
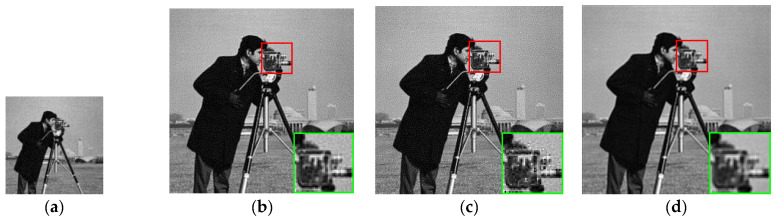

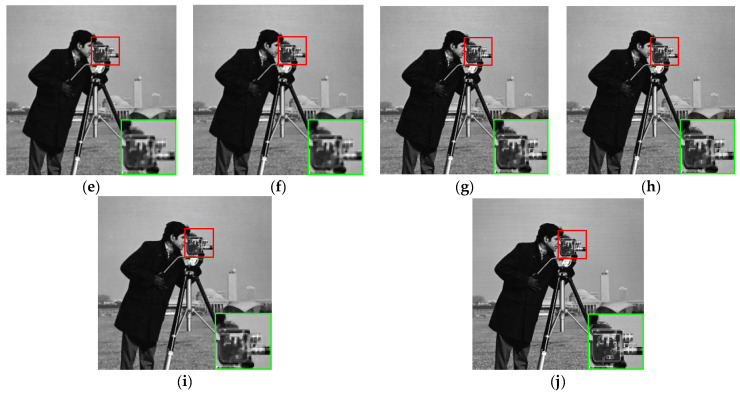
Visual comparison of MFSR algorithms for Cameraman image with mixed noises ( r=3). (**a**) the LR image. (**b**) L2 + Tikhonov. (**c**) L2 + BTV. (**d**) L1 + BTV. (**e**) BEP. (**f**) DeepSR. (**g**) IRW. (**h**) SWHQ + ABTV. (**i**) Proposed. (**j**) ground truth.

**Figure 6:**
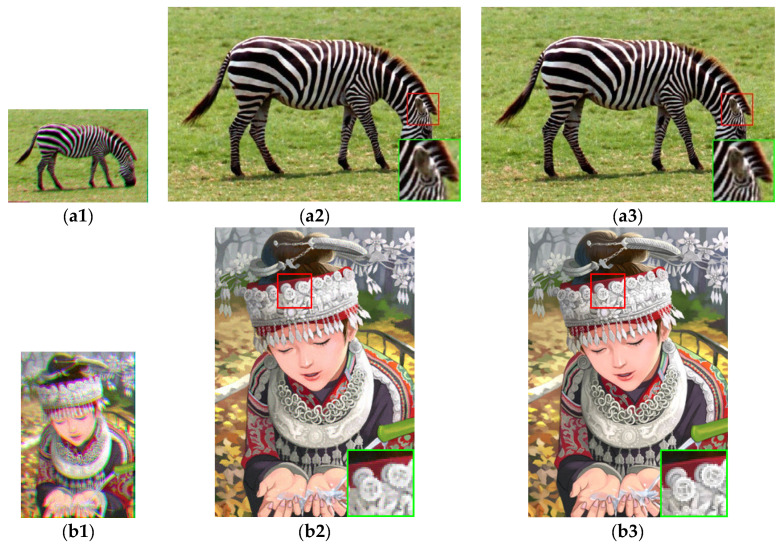

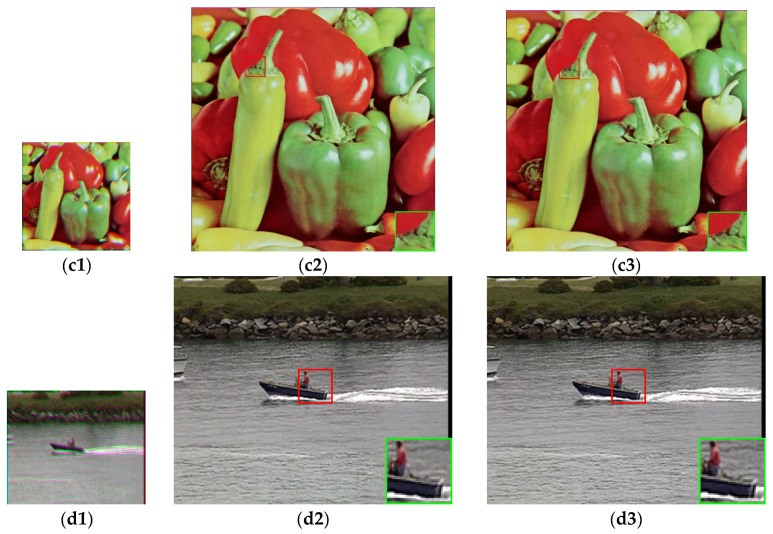
Visual comparison of MFSR algorithms with mixed noises ( r=3). (**a1**) the LR image for Zebra image. (**a2**) our method image for Zebra image. (**a3**) the ground truth image for Zebra image. (**b1**) the LR image for Comic image. (**b2**) our method image for Comic image. (**b3**) the ground truth image for Comic image. (**c1**) the LR image for Pepper image. (**c2**) our method image for Pepper image. (**c3**) the ground truth image for Pepper image. (**d1**) the LR image for Coastguard image. (**d2**) our method image for Coastguard image. (**d3**) the ground truth image for Coastguard image.

We first selected 25 appropriate LR images by using our method mentioned in Section 3.1. The results of PSNR, SSIM and IFC of these eight synthetic LR images are presented in Table 1, Table 2 and Table 3, respectively. Figure 5 shows the results of the visual comparison of several algorithms for the Cameraman image. The results of the visual comparison between our algorithm and the ground truth for some images are shown in Figure 6.

Table 1, Table 2 and Table 3 show that our proposed method in PSNR, SSIM and IFC is better than other algorithms. Our degradation model and the setting of the weight function are more reasonable. Figure 5 shows the results of the HR image estimated by these algorithms for the Cameraman image. Our algorithm performs better with image details. Figure 6 shows the comparison results of our estimated HR image and the ground truth. The result of our method is very close to the ground truth.

### 4.2. Experiments on Real Data

**Figure 7:**
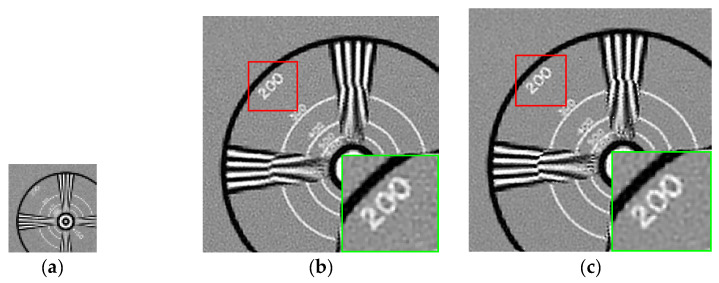

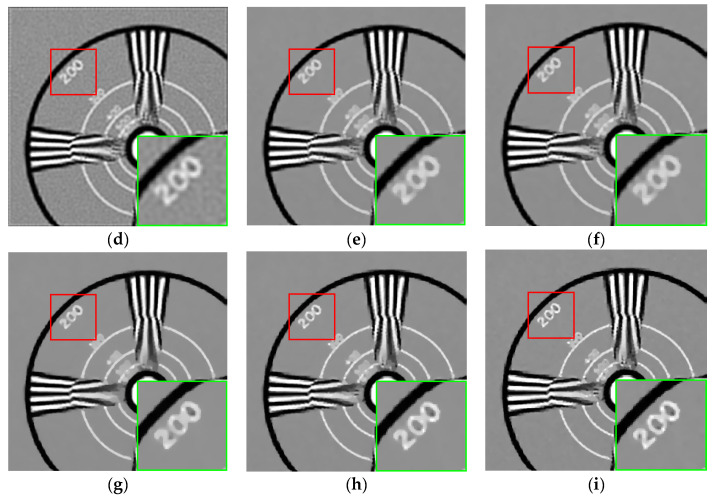
Visual comparison of MFSR algorithms for EIA frames ( r=5). (**a**) the LR image. (**b**) L2 + Tikhonov. (**c**) L2 + BTV. (**d**) L1 + BTV. (**e**) BEP. (**f**) DeepSR. (**g**) IRW. (**h**) SWHQ + ABTV. (**i**) Proposed.

**Figure 8:**
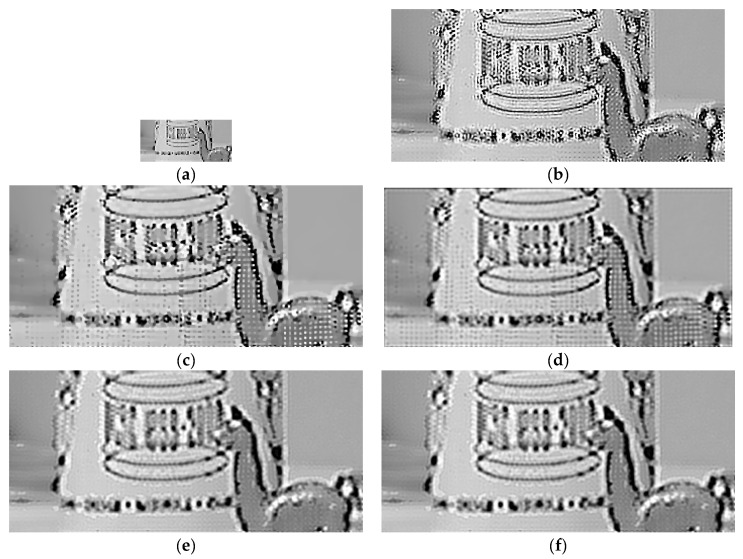

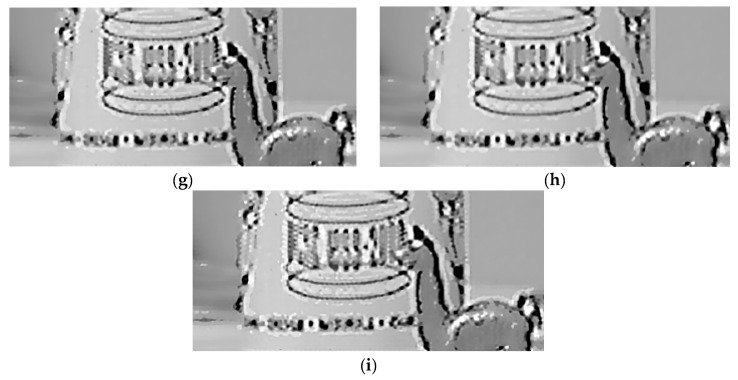
Visual comparison of MFSR algorithms for Alpaca frames (r=5). (**a**) the LR image. (**b**) L2 + Tikhonov. (**c**) L2 + BTV. (**d**) L1 + BTV. (**e**) BEP. (**f**) DeepSR. (**g**) IRW. (**h**) SWHQ + ABTV. (**i**) Proposed.

Except for synthetic data, we tested our proposed algorithm on real data which came from the Multi-Dimensional Signal Processing Research Group (MDSP) [31]. In practical applications, the image assessment matrices such as PSNR, SSIM and IFC cannot be used to evaluate the quality of the real images in the absence of ground truth images. We only tested our algorithm by visual comparison of reconstructed results for real data. Figure 7 and Figure 8 show the results of the visual comparison of EIA and Alpaca, respectively.

Figure 7 and Figure 8 show that our proposed algorithm still has better results when the magnification factor is relatively large because we employ the coarse to fine strategy which gradually enlarges to the target size. In addition, our estimated HR image, which has no obvious artifacts or noise, can maintain the details of the image and suppress the noise simultaneously, mainly because we propose the local structure tensor weighted BTV regularization term which is universal for natural images.

The experimental results on synthetic data and real data show that our proposed method has a better effect compared with current methods; especially for preserving edge information, our method has better performance.

## 5. Conclusions

MFSR is a very challenging problem. In this paper, we propose a new robust MFSR algorithm to preserve richer image detail information while suppressing noise. We first select the LR images with appropriate quality simply and quickly instead of all LR images as the input of MFSR, which reduces the computational cost of MFSR and makes it more practical. For the fidelity term, we analyze and use the half-quadratic function as the loss function. We adjust the function parameter and confidence weights adaptively according to the observation errors. We gradually reduce the observation errors of the LR images and the estimated HR image through iteration. We better suppress the noise and preserve the image edge details simultaneously using the local structure tensor weighted BTV regularization term. The combination of the local structure tensor and the sparsity of the image, as the prior knowledge of the image, can reflect the structural characteristics of the natural images.

Our proposed method is a non-blind reconstruction, which assumes that the point spread function is known. The blur kernel is often unknown in practical applications. Our next task is to study the unknown blur in MFSR. One direction of our future work is to extend our method to blind MFSR.

## Figures and Tables

**Table 1:** PSNR Results of our MFSR Algorithms on the 8 synthetic LR images under r=3.

Images	L2 + Tikhonov	L2 + BTV	L1 + BTV	BEP	DeepSR	IRW	SWHQ + ABTV	Our Proposed
Baboon	26.7519	27.6539	27.8515	28.6179	29.0428	32.0145	32.0817	**32.8181**
Barbara	26.4795	27.6776	28.0659	28.4528	28.5127	28.9558	29.0978	**29.2285**
Bridge	27.3767	27.3864	27.6665	28.5257	28.3503	28.6158	29.0555	**29.3100**
Cameraman	25.9312	28.4447	28.1831	30.3180	30.5749	31.2300	31.5659	**31.7468**
Coastguard	30.4742	33.3708	34.0352	34.1495	34.1135	34.3029	34.6220	**35.0370**
Comic	29.0123	29.2033	31.0761	31.2222	31.1298	32.0309	32.3362	**32.4921**
Pepper	31.2668	34.0678	34.3740	36.1266	36.2741	37.1517	37.2824	**37.3033**
Zebra	30.5562	34.4239	34.0824	35.0437	34.7821	35.1857	35.4068	**35.5603**
average	28.4811	30.2786	30.6668	31.5570	31.5975	32.4359	32.6810	**32.9370**

**Table 2:** SSIM Results of our MFSR Algorithms on the 8 synthetic LR images under r=3.

Images	L2 + Tikhonov	L2 + BTV	L1 + BTV	BEP	DeepSR	IRW	SWHQ + ABTV	Our Proposed
Baboon	0.8048	0.8084	0.8258	0.8482	0.8502	0.9089	0.9189	**0.9237**
Barbara	0.7624	0.7985	0.8073	0.8469	0.8475	0.8559	0.8603	**0.8712**
Bridge	0.7947	0.7994	0.8351	0.8495	0.8386	0.8551	0.8626	**0.8795**
Cameraman	0.6661	0.6961	0.7642	0.9012	0.9023	0.9049	0.9088	**0.9150**
Coastguard	0.8144	0.9075	0.9081	0.9137	0.9106	0.9277	0.9287	**0.9316**
Comic	0.8679	0.9178	0.9213	0.9458	0.9398	0.9556	0.9557	**0.9562**
Pepper	0.7168	0.8408	0.8236	0.9146	0.9138	0.9142	0.9152	**0.9158**
Zebra	0.8401	0.9241	0.9246	0.9399	0.9322	0.9426	0.9519	**0.9532**
average	0.7834	0.8366	0.8512	0.8950	0.8919	0.9081	0.9128	**0.9183**

**Table 3:** IFC Results of our MFSR Algorithms on the 8 synthetic LR images under r=3.

Images	L2 + Tikhonov	L2 + BTV	L1 + BTV	BEP	DeepSR	IRW	SWHQ + ABTV	Our Proposed
Baboon	7.3534	7.9512	7.9579	8.0944	8.4563	8.7550	8.7630	**8.8393**
Barbara	7.0135	7.3412	7.4382	8.0463	8.3758	8.6110	**8.7294**	8.7274
Bridge	5.9417	6.3412	6.7888	7.1622	6.9875	7.2175	7.2446	**7.3342**
Cameraman	4.1673	4.3269	4.3504	4.9557	4.9876	5.0998	5.1277	**5.1951**
Coastguard	6.0716	6.2598	6.2280	6.3565	6.1789	6.7285	6.8523	**6.8968**
Comic	7.8456	8.5884	8.5808	9.0407	8.9132	9.5569	9.6000	**9.6259**
Pepper	4.5122	4.8135	4.7396	5.7484	5.7478	5.7484	5.7926	**5.8216**
Zebra	8.0852	8.3143	8.5088	8.8699	8.7891	9.0015	9.0662	**9.1005**
average	6.3738	6.7421	6.8241	7.2843	7.3045	7.5898	7.6470	**7.6926**

## Data Availability

Datasets available online: https://users.soe.ucsc.edu/~milanfar/software/sr-datasets.html (accessed on 4 March 2021).

## References

[1] Zhang H., Zhang L., Shen H. (2012). A super-resolution reconstruction algorithm for hyperspectral images. Signal Process..

[2] Köhler T., Brost A., Mogalle K., Zhang Q., Köhler C., Michelson G., Hornegger J., Tornow R.P. Multi-frame super-resolution with quality self-assessment for retinal fundus videos. Proceedings of the International Conference on Medical Image Computing and Computer-Assisted Intervention.

[3] Zhang L., Zhang H., Shen H., Li P. (2010). A super-resolution reconstruction algorithm for surveillance images. Signal Process..

[4] Tsaiand R.Y., Huang T.S. (1984). Multi-frame image restoration and registration. Adv. Comput. Vis. Image Process..

[5] Takeda H., Farsiu S., Milanfar P. (2007). Kernel regression for image processing and reconstruction. IEEE Trans. Image Process..

[6] Baatz M., Eichenseer A., Kaup A. Multi-image super resolution using a dual weighting scheme based on voronoi tessellation. Proceedings of the 2016 IEEE International Conference on Image Processing (ICIP).

[7] Farsiu S., Robinson M.D., Elad M., Milanfar P. (2004). Fast and robust multiframe super resolution. IEEE Trans. Image Process..

[8] Zeng X., Yang L. (2013). A robust multiframe super-resolution algorithm based on half-quadratic estimation with modified BTV regularization. Digit. Signal Process..

[9] Köhler T., Huang X., Schebesch F., Aichert A., Maier A., Hornegger J. (2016). Robust Multiframe Super-Resolution Employing Iteratively Re-Weighted Minimization. IEEE Trans. Comput. Imaging.

[10] Liu X., Chen L., Wang W., Zhao J. (2018). Robust Multi-Frame Super-Resolution Based on Spatially Weighted Half-Quadratic Estimation and Adaptive BTV Regularization. IEEE Trans. Image Process..

[11] Wang P., Hu X., Xuan B., Mu J. Super resolution reconstruction via multiple frames joint learning. Proceedings of the 2011 International Conference on Multimedia and Signal Processing (ICMSP).

[12] Kato T., Hino H., Murata N. (2015). Multi-frame image super resolution based on sparse coding. Neural Netw..

[13] Liao R., Tao X., Li R., Ma Z., Jia J. Video super-resolution via deep draft-ensemble learning. Proceedings of the IEEE International Conference on Computer Vision.

[14] Noor D.F., Li L., Li Z., Bhattacharyya S. Multi-frame super resolution with deep residual learning on flow registered non-integer pixel images. Proceedings of the 2019 IEEE International Conference on Image Processing (ICIP).

[15] Huang Y., Wang W., Wang L. (2015). Bidirectional recurrent convolutional networks for multi-frame super-resolution. Adv. Neural Inf. Process. Syst..

[16] Huang Y., Wang W., Wang L. (2018). Video super-resolution via bidirectional recurrent convolutional networks. IEEE Trans. Pattern Anal. Mach. Intell..

[17] Patanavijit V., Jitapunkul S. A robust iterative multiframe super resolution reconstruction using a Huber Bayesian approach with Huber Tikhonov regularization. Proceedings of the IEEE International Symposium on Intelligent Signal Processing and Communications.

[18] Patanavijit V., Jitapunkul S. (2007). A Lorentzian stochastic estimation for a robust iterative multiframe super-resolution reconstruction with Lorentzian–Tikhonov regularization. J. Adv. Signal Process..

[19] Anastassopoulos A., Vassilis P. (2009). Regularized super-resolution image reconstruction employing robust error norms. Opt. Eng..

[20] Yue L., Shen H., Yuan Q., Zhang L. (2014). A locally adaptive L1-L2 norm for multi-frame super-resolution of images with mixed noise and outliers. Signal Process..

[21] Elad M., Feuer A. (1997). Restoration of a single super resolution image from several blurred, noisy, and undersampled measured images. IEEE Trans. Image Process..

[22] Pickup L.C., Capel D.P., Roberts S.J., Zisserma A.N. (2007). Overcoming registration uncertainty in image super-resolution: Maximize or marginalize. EURASIP J. Adv. Signal Process..

[23] Traonmilin Y., Ladjal S., Almansa A. On the amount of regularization for superresolution interpolation. Proceedings of the 2012 20th European Signal Processing Conference (EUSIPCO).

[24] Gu K., Zhai G., Lin W., Yang X., Zhang W. (2015). No-reference image sharpness assessment in autoregressive parameter space. IEEE Trans. Image Process..

[25] Gu K., Li L., Lu H., Min X., Lin W. (2017). A fast reliable image quality predictor by fusing micro-and macro-structures. IEEE Trans. Ind. Electron..

[26] Rahimi A., Moallem P., Shahtalebi K., Momeni M. (2018). Preserving quality in minimum frame selection within multi-frame super-resolution. Digit. Signal Process..

[27] Charbonnier L., Blanc-Féraud G., Aubert M. (1997). Barlaud Deterministic edge-preserving regularization in computed imaging. IEEE Trans. Image Process..

[28] Scales J.A., Gersztenkorn A. (1988). Robust methods in inverse theory. Inverse Probl..

[29] Nabney I.T. (2002). NETLAB: Algorithms for Pattern Recognition.

[30] Zeyde R., Elad M., Protter M. (2010). On single image scale-up using sparse-representations. Proceedings of the International Conference on Curves and Surfaces.

[31] Farsiu S. (2017). MDSP Super-Resolution and Demosaicing Datasets. https://users.soe.ucsc.edu/~milanfar/software/sr-datasets.html.

